# Comparison of Different Risk-Stratification Systems for the Diagnosis of Benign and Malignant Thyroid Nodules

**DOI:** 10.3389/fonc.2019.00378

**Published:** 2019-05-14

**Authors:** Yan Shen, Miao Liu, Jie He, Shu Wu, Ming Chen, Yonglin Wan, Linjun Gao, Xiaoyan Cai, Jun Ding, Xiaohong Fu

**Affiliations:** ^1^Department of Medical Ultrasound, Gong Li Hospital, Shanghai, China; ^2^Department of Surgery, Gong Li Hospital, Shanghai, China; ^3^Department of Pathology, Gong Li Hospital, Shanghai, China

**Keywords:** thyroid nodule, ultrasonography, diagnosis, risk of malignancy, risk-stratification systems

## Abstract

**Introduction:** To compare the efficacy of four different ultrasound-based risk-stratification systems in assessing the malignancy risk of thyroid nodules in the Chinese population.

**Methods:** We retrospectively reviewed the digital ultrasound images of 1,568 patients (1,612 thyroid nodules) who underwent surgery in our hospital between January 2012 and December 2017. All thyroid nodules were pathologically identified as malignant or benign. We evaluated the following ultrasound characteristics: size, location, composition, echogenicity, shape, margins, calcification or echogenic foci, and extrathyroidal extension. Each nodule was categorized using four risk-stratification systems: the American Thyroid Association (ATA) classification, the Thyroid Imaging, Reporting, and Data System (TIRADS) of the American College of Radiology (ACR-TIRADS), the European Thyroid Association TIRADS (EU-TIRADS), and the TIRADS developed by Kwak et al. (Kwak-TIRADS). The diagnostic performance of each risk-stratification system relative to the pathological results was analyzed. We used receiver operating characteristic curves to identify cutoff values that yielded optimal sensitivity (SEN), specificity (SPE), positive predictive value (PPV), negative predictive value (NPV), and accuracy (ACC).

**Results:** Of the 1,612 nodules, 839 (52.0%) were benign, and 773 (48.0%) were malignant. The AUCs of the ACR-TIRADS, EU-TIRADS, Kwak-TIRADS, and ATA classification were 0.879, 0.872, 0.896, and 0.869, respectively. The Kwak-TIRADS had the best SEN, NPV, ACC, and AUC, while the ACR-TIRADS had the best SPE and PPV.

**Conclusion:** All four risk-stratification systems had good diagnostic performances (AUCs > 86%). Considering its high SEN, NPV, ACC, and AUC, we believe that the Kwak-TIRADS may be the more effective risk-stratification system in the Chinese population.

## Introduction

Thyroid nodules are very common with ultrasound detection rates of 50–60% (1). However, the detection rates of malignant nodules are significantly lower at 5–15% (2). Ultrasonography is the primary modality used for imaging thyroid nodules, as it is readily accessible, noninvasive, and cost-effective (3). Ultrasound-guided fine-needle aspiration cytology (US-FNAC) is the most effective and practical technique to determine whether a thyroid nodule is malignant or whether surgery is required to establish a definitive diagnosis (4). Due to the complex imaging features of thyroid nodules, several distinct risk-stratification systems have been developed to standardize the diagnostic procedure. The risk-stratification systems commonly used to classify thyroid nodules are (1) the American Thyroid Association (ATA) classification, (2) the Thyroid Imaging, Reporting, and Data System (TIRADS) developed by the American College of Radiology (the ACR-TIRADS), (3) the TIRADS published by the European Thyroid Association (the EU-TIRADS), (4) the TIRADS developed by Kwak et al. (the Kwak-TIRADS), and (5) the British Thyroid Association classification. These five risk-stratification systems are based on a comprehensive analysis of multiple ultrasonographic features, and enable the stratification of the malignancy risk of thyroid nodules and provide a basis for determining the necessity of US-FNA.

More than 90% of thyroid carcinomas are well-differentiated papillary thyroid carcinomas, which are associated with a low malignancy potential, good prognosis, and excellent 5-year survival rates of 95–97% (5, 6). Therefore, early diagnosis is particularly important in the treatment of thyroid nodules. Due to several advancements in ultrasound technology, such as the development of elastography and contrast-enhanced ultrasonography, the diagnostic accuracy of ultrasonography in patients with thyroid nodules is increasing (7, 8). However, conventional ultrasonography remains the most widely employed diagnostic tool for detecting for thyroid nodules due to its wide availability. The purpose of this study was to compare the four risk-stratification systems used in our research center, namely, the ATA classification, the ACR-TIRADS, the EU-TIRADS, and the Kwak-TIRADS, in terms of their efficacy in determining the malignancy risk of thyroid nodules. Our findings will help provide a theoretical basis for the selection of the optimal risk-stratification system.

## Methods

### Ethics and Consent

This retrospective study was approved by the institutional review board of our hospital. Informed consent was waived for this retrospective review.

### Patients

This study involved all patients with thyroid nodules who underwent surgery in our hospital between January 2012 and December 2017. Patients were eligible for inclusion in this study if they were between 18 and 80 years of age with nodules measuring more than 5 mm in diameter, as nodules measuring <5 mm have no clinical significance (5). US-FNAC was introduced in our hospital in February 2015. Thus, the indication of thyroid surgery was based on the US-FNAC findings after February 2015. Prior to this time, surgery was considered to be indicated for nodules that showed at least two ultrasound features that were highly suggestive of malignancy and for nodules that appeared to be benign but were associated with clinical symptoms.

Patients with histories of invasive procedures, such as ablation or FNA, those without complete ultrasonographic data, and those with any mismatch between the ultrasound images and the pathological results were excluded from this study.

### Conventional Ultrasonography

Real-time ultrasound examinations were performed using the iU22 device (Philips Medical Systems, Bothell, WA, USA; 5–12 MHz linear probe) or the S3000 device (Siemens Medical Solutions, Mountain View, CA, USA; 5–14 MHz linear probe) by five radiologists with more than 7 years of experience each in thyroid ultrasonography. Ultrasonography was performed with the patient in a supine position and the neck slightly extended. The probe was placed on the surface of the neck with slight pressure. The entire thyroid gland was scanned first to determine the echo structure of the thyroid parenchyma. When nodules were detected, they were placed in the center of the screen for analysis. Machine settings such as gain, depth, focus, and dynamic range were adjusted as necessary to achieve high-quality ultrasonographic images. The ultrasound data were recorded and stored for further analysis.

### Image Evaluation

Two radiologists (YS and ML) who did not participate in the image capture independently reviewed, analyzed, and classified the imaging data. They have 11 and 15 years of experience in thyroid ultrasonography, respectively. They were blinded to the patients' medical information, including previous imaging and pathological results. A basic consensus on the lexicon for the four guidelines had previously been reached, and included imaging characteristics such as location, composition, echogenicity, shape, margin, calcification or echogenic foci, and neck lymph nodes (3, 9–11).

The locations were divided into right, left, and isthmus. Specific descriptions were used to ensure consistency between the surgical and pathological nodules. Each nodule was described as being located in the upper, middle, or lower third of the thyroid gland, and close to the anterior capsule, in the middle of the thyroid gland, or close to the posterior capsule. The composition was described as cystic or almost completely cystic, spongiform, mixed cystic and solid, or solid or almost completely solid. Echogenicity was determined relative to the surrounding glands and was described as anechoic, hyperechoic, isoechoic, hypoechoic, or very hypoechoic (lower echogenicity than that of the adjacent strap muscle). The shape was classified as wider-than-tall and taller-than-wide. Margins were classified as smooth, ill-defined, lobulated or irregular, or extrathyroidal extension. Calcification or echogenic foci were classified as none or large comet-tail artifacts (V-shaped, >1 mm, cystic components), macrocalcification (>1 mm), peripheral (rim) calcification, or punctate echogenic foci or microcalcification. The lymph node status was defined as normal or metastatic.

### Statistical Analysis

The SPSS software (version 19.0, SPSS Inc., Chicago, IL, USA) and MedCalc software (version 15.8, Mariakerke, Belgium) were used for statistical analysis. Continuous variables were expressed as mean ± standard deviation or as ranges. Classification data were compared using the chi-square test or the Fisher exact test, while continuous variables were compared using the independent-samples *t*-test. The receiver-operating characteristic (ROC) curve was used to comparatively analyze the diagnostic value of the four guidelines. The areas under the curve (AUCs) of the diagnostic ability of the four risk-stratification systems were calculated, and the Cochran *Q*-test and *z*-test were used for statistical analysis. The best cutoff values were obtained from the ROC analyses, and the corresponding sensitivity (SEN), specificity (SPE), positive predictive value (PPV), negative predictive value (NPV), and accuracy (ACC) were calculated. Two-sided *P* < 0.05 were considered to indicate statistical significance.

## Results

### General Characteristics

During the study period, a total of 1,634 patients with 1,687 thyroid nodules underwent surgery in our hospital. The indication of surgery was based on the US-FNAC findings in the case of 757 nodules. After the application of the selection criteria, 1,568 patients with 1,612 nodules were enrolled into this study. Of these patients, 1,156 were women (1,192 nodules) with a mean age of 51 ± 12 years (range, 18–80 years), and 412 were men (420 nodules) with a mean age of 49 ± 11 years (range, 18–78 years). Of the 1,612 thyroid nodules, 839 (52.05%) were diagnosed as benign on pathological examination (nodular goiter, 525; adenoma, 213; Hashimoto thyroiditis, 73; and subacute thyroiditis, 28). The other 773 (47.95%) nodules were diagnosed as malignant on pathological examination (papillary carcinoma, 738; follicular carcinoma, 23; medullary carcinoma, 10; and undifferentiated carcinoma, 2).

### Ultrasonographic Predictors of Malignancy

**Figure 1:**
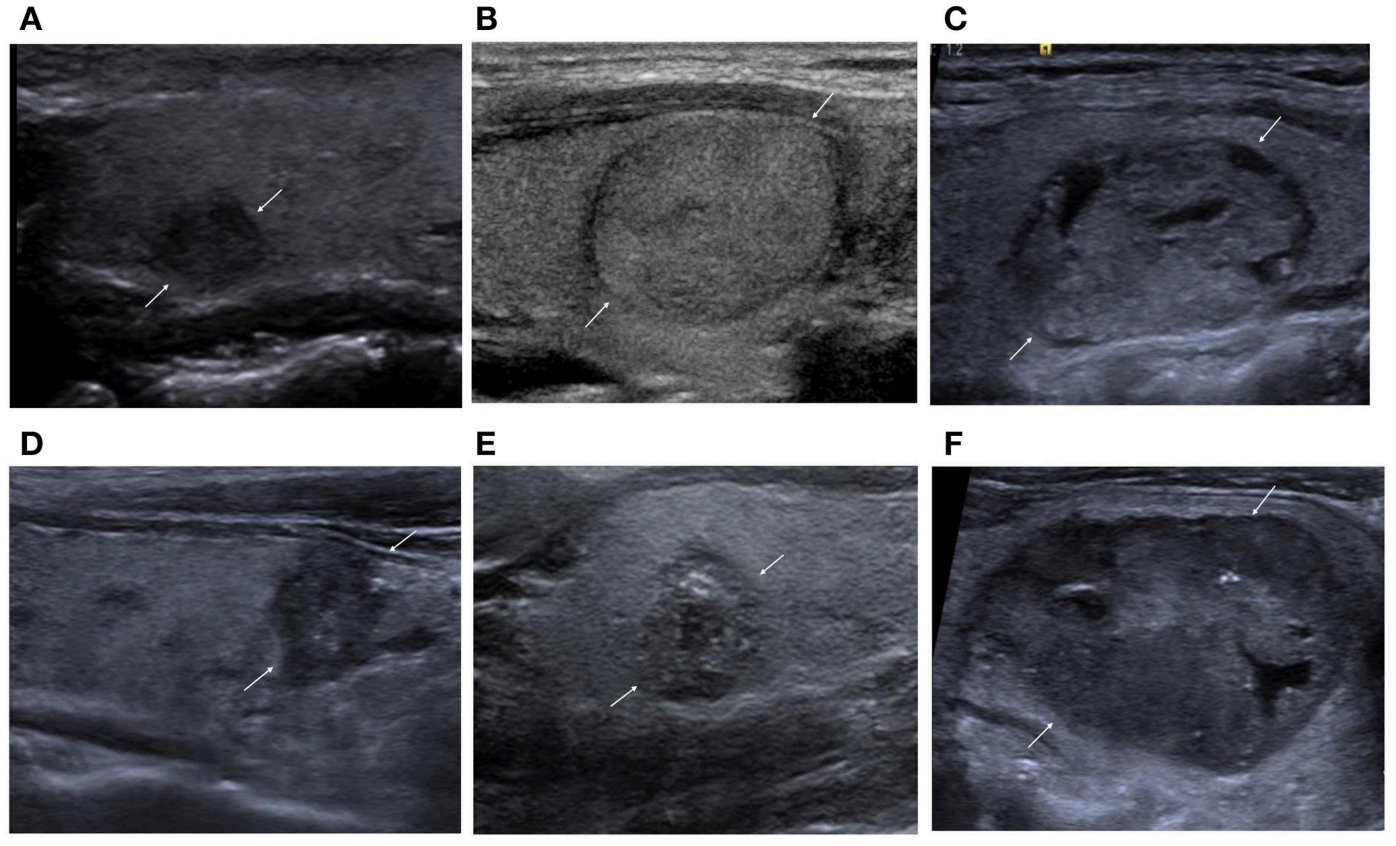
**(A)** Nodular goiter. Solid and hypoechoic nodule. ACR-TIRADS, 4; EU-TIRADS, 4; Kwak-TIRADS, 4B; ATA classification, high suspicion. **(B)** Follicular adenoma. Solid and isoechoic nodule. ACR-TIRADS, 3; EU-TIRADS, 3; Kwak-TIRADS, 4A; ATA classification, low suspicion. **(C)** Follicular adenoma. Mixed cystic and solid. ACR-TIRADS, 3; EU-TIRADS, 3; Kwak-TIRADS, 3; ATA classification, low suspicion. **(D)** Papillary thyroid carcinoma. Solid and hypoechoic nodule with taller-than-wide shape, irregular margins, and microcalcification. ACR-TIRADS, 5; EU-TIRADS, 5; Kwak-TIRADS, 4C; ATA classification, high suspicion. **(E)** Papillary thyroid carcinoma. Solid and hypoechoic nodule with microcalcification and macrocalcification. ACR-TIRADS, 5; EU-TIRADS, 5; Kwak-TIRADS, 4C; ATA classification, high suspicion. **(F)** Medullary thyroid carcinoma. Mostly solid and hypoechoic nodule with dispersed microcalcifications. ACR-TIRADS, 5; EU-TIRADS, 5; Kwak-TIRADS, 4C; ATA classification, high suspicion.

The malignant nodules were significantly smaller than the benign nodules (13.58 ± 11.00 mm vs. 19.69 ± 11.57 mm; *P* < 0.001). In addition, patients with malignant nodules were significantly younger than those with benign nodules (48 ± 13 years vs. 53 ± 12 years; *P* < 0.001). No significant sex-related differences were observed between patients with benign and malignant nodules, with similar female-to-male ratios in the benign (2.94, 626/213) and malignant (2.73, 566/207) groups (*P* = 0.479). Compared to the benign nodules, the malignant nodules were significantly more likely to have a solid or mostly solid composition, hypoechogenicity or very hypoechogenicity, taller-than-wide shape, lobulated or irregular margins, extrathyroidal extension, microcalcifications, and lymph node metastasis (*P* < 0.05 for all; Table 1 and Figure 1).

**Table 1:** Summary of demographic and ultrasonographic features.

**Parameter**	**Pathological result**		**Total**	***P*-value**
	**Benign**	**Malignant**		
No. of nodules	*n* = 839	*n* = 773	*n* = 1,612	
Age, years				<0.01
Mean	53 ± 12	48 ± 13		
Range	18–80	22–79		
Gender, n (%)				0.479
Male	213 (25.4)	207 (26.8)	420	
Female	626 (74.6)	566 (73.2)	1,192	
Tumor size (mm)				<0.01
Mean	19.69 ± 11.58	13.59 ± 11.01		
Range	6–84	6–120		
Composition, *n* (%)				<0.01
Cystic or almost completely cystic	7 (0.9)	1 (0.1)	8	
Mixed cystic and solid	315 (37.5)	60 (7.8)	375	
Solid or almost solid	517 (61.6)	712 (92.1)	1,229	
Echogenicity, *n* (%)				<0.01
Anechoic	17 (2.1)	1 (0.1)	18	
Hyperechoic or isoechoic	68 (8.1)	13 (1.7)	81	
Hypoechoic	736 (87.7)	714 (92.4)	1,450	
Very hypoechoic	18 (2.1)	45 (5.8)	63	
Shape, *n* (%)				<0.01
Wider-than-tall	803 (95.7)	441 (57.1)	1,244	
Taller-than-wide	36 (4.3)	332 (42.9)	368	
Margins, *n* (%)				<0.01
Smooth	787 (93.8)	457 (59.1)	1,244	
vIll-defined	13 (1.5)	73 (9.4)	86	
Lobulated or irregular	39 (4.7)	201 (26.0)	240	
Extrathyroidal extension	0 (0.0)	42 (5.5)	42	
Calcification or echogenic foci, *n* (%)				<0.01
None or large comet-tail artifacts	630 (75.1)	206 (26.7)	836	
Macrocalcification	87 (10.4)	27 (3.5)	114	
Peripheral (rim) calcification	26 (3.0)	38 (4.9)	64	
Microcalcification	96 (11.5)	502 (64.9)	598	
Lymph node metastasis, *n* (%)				<0.01
Normal	839 (100.0)	692 (89.5)	1,531	
Metastasis	0 (0.0)	81 (10.5)	81	

*Data in parentheses are percentages*.

### Malignancy Risk Stratification

The risk of malignancy significantly differed among the four risk-stratification systems (*P* < 0.05; Table 2). The malignancy risk was within the recommended range in the case of all guidelines, except for the ATA classification, in which case the risk was too low.

**Table 2:** Comparison of malignancy rates among the four guidelines.

**Scoring system and category**	**Characteristic**	**Final diagnosis**		**Recommended malignancy risk (%)**	**Calculated malignancy risk (%)**	***P*-value**
		**Benign (*n* = 839)**	**Malignant (*n* = 773)**			
ACR-TIRADS						<0.001
2	Not suspicious	6 (0.7)	0 (0.0)	<2	0	
3	Mildly suspicious	269 (32.1)	13 (1.7)	5	4.6	
4	Moderately suspicious	459 (54.7)	78 (10.1)	5–20	14.5	
5	Highly suspicious	105 (12.5)	682 (88.2)	>20	86.7	
EU-TIRADS						<0.001
2	Benign	8 (0.9)	0 (0.0)	~0	0	
3	Low risk	269 (32.1)	11 (1.4)	2–4	3.9	
4	Intermediate risk	402 (47.9)	41 (5.3)	6–17	9.3	
5	High risk^&^	160 (19.1)	721 (93.3)	26–87	81.8	
Kwak-TIRADS						<0.001
2	Benign	3 (0.3)	0 (0.0)	0	0	
3	No suspicious feature	250 (29.8)	6 (0.8)	2.0–2.8	2.3	
4A	1 suspicious US feature	106 (12.6)	7 (0.9)	3.6–12.7	6.1	
4B	2 suspicious US features	364 (43.4)	39 (5.0)	6.8–37.8	9.6	
4C	3 or 4 suspicious US features	114 (13.6)	683 (88.4)	21–91.9	85.6	
5	5 suspicious US features^#^	2 (0.3)	38 (4.9)	88.7–97.9	95.0	
ATA classification						<0.001
Does not meet criteria	Benign	5 (0.6)	0 (0.0)	0	0	
Very low suspicion	Spongiform or partially cystic nodule	187 (22.3)	6 (0.8)	<3	3.1	
Low suspicion	Hyper-to-isoechoic solid or partially cystic nodules with uniform solid areas	149 (17.8)	17 (2.2)	5–10	10.2	
Intermediate suspicion	Hypoechoic, solid nodules with smooth margins	348 (41.5)	42 (5.4)	10–20	10.8	
High suspicion	Solid hypoechoic nodule or solid hypoechoic component in a partially cystic nodule with one or more suspicious US features^*^	150 (17.8)	708 (91.6)	70–90	82.5	

*Data are shown as numbers (percentages) unless otherwise indicated*.

*ACR, 2017 American College of Radiology guidelines; EU, 2017 European Thyroid Association Guidelines; (9) TIRADS developed by Kwak et al.; ATA, 2015 American Thyroid Association Management Guidelines for Adult Patients with Thyroid Nodules and Differentiated Thyroid Cancer; US, ultrasound*.

& *At least one of the following features indicating a high suspicion of malignancy: irregular shape, irregular margin, microcalcification, marked hypoechogenicity (and solid lesion)*.

# *Solid composition, hypoechogenicity or marked hypoechogenicity, microlobulated or irregular margin, microcalcification, taller-than-wide shape*.

* *Irregular margin, microcalcification, taller-than-wide shape, disrupted rim calcification with hypoechoic soft-tissue component, extrathyroidal extension*.

### Diagnostic Cutoffs

**Figure 2:**
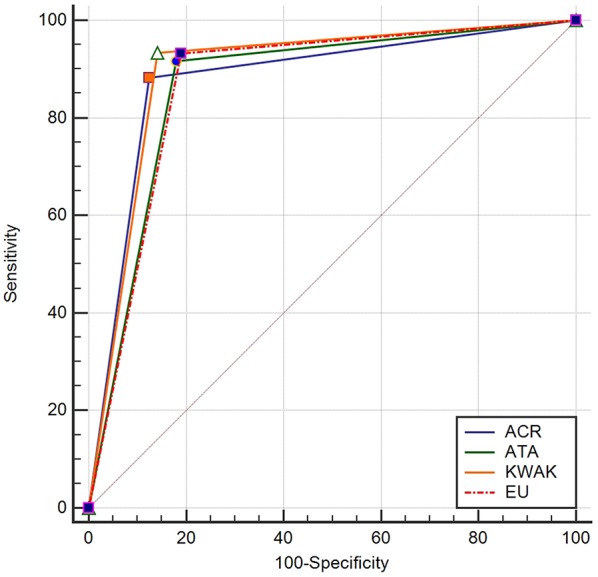
Analysis of the receiver operating characteristic curves of the four guidelines.

The cutoff value of the ACR-TIRADS was TIRADS 5, whose SEN, SPE, PPV, NPV, ACC, and AUC were 88.2, 87.4, 86.7, 87.5, 87.8, and 87.9%, respectively. The cutoff value of the EU-TIRADS was TIRADS 5, and its SEN, SPE, PPV, NPV, ACC, and AUC were 93.37, 81.05, 81.9, 92.9, 86.9, and 87.2%, respectively. The cutoff value of the Kwak-TIRADS was TIRADS 4C, whose SEN, SPE, PPV, NPV, ACC, and AUC were 93.5, 85.8, 86.0, 93.4, 89.5, and 89.6%, respectively. The cutoff value of the ATA classification was “highly suspicious,” and its SEN, SPE, PPV, NPV, ACU, and AUC were 91.7, 82.0, 82.4, 92.8, 86.7, and 86.9%, respectively. The Kwak-TIRADS had the highest SEN, PPV, ACC, and AUC, while the ACR-TIRADS had the highest SPE and NPV (Table 3 and Figure 2).

**Table 3:** Diagnostic efficacy of the four guidelines.

**Parameter**	**Cutoff value**	**SEN (%)**	**SPE (%)**	**PPV (%)**	**NPV (%)**	**ACC (%)**	**AUC (%)**	**95% CI (%)**
ACR-TIRADS	5	88.2	87.5	86.7	89.0	87.8	87.9	86.0–89.7
EU-TIRADS	5	93.4	81.1	81.9	92.9	86.9	87.2	85.3–89.0
Kwak-TIRADS	4C	93.5	85.8	86.0	93.4	89.5	89.6	87.8–91.3
ATA classification	High suspicion	91.7	82.0	82.4	92.9	86.7	86.9	85.0–88.8

*ACR, 2017 American College of Radiology guidelines; EU, 2017 European Thyroid Association Guidelines; (9) TIRADS developed by Kwak et al.; ATA, 2015 American Thyroid Association Management Guidelines for Adult Patients with Thyroid Nodules and Differentiated Thyroid Cancer; SEN, sensitivity; SPE, specificity; PPV, positive predictive value; NPV, negative predictive value; ACU, accuracy; AUC, area under the curve; 95% CI, confidence interval*.

The Cochran *Q* test revealed differences among the four systems (Cochran *Q* = 150.29, *P* < 0.01). The AUC of the Kwak-TIRADS significantly differed from those of the other three systems (*z*-values: 3.405 for ACR, 5.748 for ATA, and 5.485 for EU; *P* < 0.01 for each). No significant differences were detected among the other three risk-stratification systems (Supplementary Table S1).

### Diagnostic Efficacy According to Nodule Size

We divided the nodules into three groups based on their diameter: ≤10 mm, >10 mm but ≤20 mm, and >20 mm. For nodules with diameters of ≤10 mm, the ACR-TIRADS had the greatest AUC. For nodules with diameters >10 mm, the Kwak-TIRADS had the greatest AUC. We found that the diagnostic efficacy of the four guidelines varied with nodule size and that the efficacy was higher for larger nodules (Table 4).

**Table 4:** Diagnostic efficacy of the four guidelines for different-sized nodules.

**Size**	**Parameter**	**SEN (%)**	**SPE (%)**	**PPV (%)**	**NPV (%)**	**ACU (%)**	**AUC (%)**	**95% CI**
≤10 mm	ACR	95.3	66.5	86.7	86.1	86.6	81.1	76.7–85.6
	EU	97.7	61.1	85.2	91.9	86.6	79.4	74.7–84.0
	Kwak	97.4	64.7	86.2	91.5	87.3	80.8	76.3–85.4
	ATA	96.4	61.1	85.1	87.9	85.7	78.6	74.0–83.3
>10 mm and ≤20 mm	ACR	81.9	88.0	83.3	87.0	85.4	85.1	81.6–88.6
	EU	88.0	80.4	76.6	90.1	83.6	84.2	80.8–87.7
	Kwak	88.4	86.2	82.4	91.0	87.1	87.3	84.1–90.5
	ATA	86.8	81.5	77.4	89.4	83.7	84.4	81.0–87.9
>20 mm	ACR	79.7	97.6	93.0	92.2	92.4	88.6	84.9–93.3
	EU	91.0	91.8	81.8	96.2	97.4	91.4	88.1–94.9
	Kwak	91.7	96.3	91.0	96.6	95.0	93.9	91.1–97.2
	ATA	88.0	93.0	83.6	95.0	91.5	90.5	86.9–94.2

*ACR, 2017 American College of Radiology guidelines; EU, 2017 European Thyroid Association Guidelines; (9) TIRADS developed by Kwak et al.; ATA, 2015 American Thyroid Association Management Guidelines for Adult Patients with Thyroid Nodules and Differentiated Thyroid Cancer; SEN, sensitivity; SPE, specificity; PPV, positive predictive value; NPV, negative predictive value; ACU, accuracy; AUC, area under the curve; 95% CI, confidence interval*.

## Discussion

Ultrasonography is currently the preferred method for evaluating thyroid nodules (2). Ultrasound features such as hypoechoic or very hypoechoic, taller-than-wide, microcalcifications, and irregular margins are associated with malignancy (12). However, single images are unreliable in predicting the malignancy risk of thyroid nodules (13). Therefore, researchers have developed ultrasound models that combine several ultrasound features in order to improve the diagnostic performance of ultrasonography for thyroid nodules.

In recent years, many distinct TIRADS guidelines have applied ultrasound features to classify thyroid nodules as malignant or benign, or to recommend US-FNA (3, 9, 11, 14–21). Such diagnostic standards not only clarify the malignancy risk of thyroid nodules but also help guide treatment. Many versions of TIRADS were modeled on the BIRADS, which has been widely used in breast cancer diagnoses. For instance, the Kwak-TIRADS, a simplified classification based on five malignant features, has proved to be clinically useful and accessible (22). However, independent risk factors are not weighted in the Kwak-TIRADS guidelines. For example, microcalcification, which carries a higher malignancy risk than solid consistency or hypoechoic appearance, has been deemed an equal indicator of malignancy risk. In addition, extrathyroidal extension, an important risk factor, has not been included. Despite concerted efforts, no TIRADS classification has been widely accepted, especially in the United States. In 2015 and 2017, two versions of the ACR-TIRADS white paper were published (11, 23). The difference between them was that in the 2017 version, nodule size was a criterion for US-FNA but not for malignancy risk stratification. The ACR-TIRADS is suitable for all nodules, as it integrates all ultrasonographic characteristics, which are scored from 0 to 3 based on their malignant potential. The higher the score, the higher the malignancy risk. Therefore, the ACR-TIRADS is an objective and comprehensive method to evaluate the characteristics of each thyroid nodule and also to guide therapy. The disadvantage is that it is more complicated than the other guidelines. Moreover, malignant nodules with mixed echo patterns are scored lower in the ACR-TIRADS, resulting in misdiagnosis. The risk of malignancy for ACR-TIRADS 5 is ≥20%.

The EU-TIRADS is based on a review of the literature and on the American Association of Clinical Endocrinologists, the ATA, and Korean guidelines (3). The EU-TIRADS is similar to the ACR-TIRADS but is simpler. The following characteristics indicate a high risk of malignancy under the EU-TIRADS guidelines: irregular shape, irregular margins, microcalcifications, and marked hypoechogenicity. This system classifies mildly hypoechoic nodules into four categories. For category-4 nodules that measure >1.5 cm, the EU-TIRADS recommends FNA, which is an excellent recommendation for thyroid adenomas and adenocarcinomas. However, the EU-TIRADS does not include solidity as an independent risk factor and only considers hypoechogenicity.

The ATA guidelines were first published in 2009 and revised in 2015 (13). The ultrasonic signature of increased nodular vascularity was removed from the 2015 guidelines. In addition, the risk associated with hypoechogenicity was reduced. The ATA guidelines clearly identified three characteristics that are highly indicative of malignant nodules (median, >90%): microcalcifications, irregular edges, and taller-than-wide shape. These guidelines directly push forward the concept of risk stratification. However, the ATA classification has been developed for differentiated thyroid cancer in adults. In this study, we applied the ATA guidelines to the two cases of undifferentiated thyroid cancers, which were classified as highly suspicious nodules. The drawback of the ATA guidelines, like the EU-TIRADS, is the use of risk stratification to classify suspicious ultrasound features of different significance into the same hierarchy, with no independent categorization of solidity as an independent risk factor.

In this study, the AUCs of the four methods were more than 86%, indicating that all of them had good diagnostic performance. Some benign nodules were misclassified as malignant nodules. In this study, 28 subacute thyroiditis nodules and 24 Hashimoto thyroiditis nodules were misidentified as malignant because of their solid composition, hypoechoic or very hypoechoic appearance, and taller-than-wide shape. However, some of these lesions may be correctly diagnosed on the basis of the clinical history, thyroid-function indicators, and results of other newer technologies such as ultrasound elastography. It should be noted that all 28 subacute thyroiditis nodules that were misidentified as malignant were examined before February 2015. After this time, similar nodules were not misdiagnosed because of the use of US-FNAC. A total of 96 benign solid hypoechoic nodules were misdiagnosed as malignant nodules because they had microcalcifications or both macro- and microcalcifications. Eight benign nodules were misdiagnosed because of their solidity, irregular margins, and mixed echogenicity.

Similarly, some malignant nodules were mislabeled as benign nodules. Small nodules, such as thyroid microcarcinomas, with diameters of 6–10 mm do not exhibit the characteristic malignant features and were mistaken for benign lesions. In addition, some malignant nodules were classified as benign due to cystic degeneration and the lack of other malignant characteristics. In this study, ultrasonography and US-FNAC helped in the selection of surgical procedures. US-FNAC could differentiate most benign and malignant nodules. Benign nodules were commonly treated using lobectomy or hemithyroidectomy. The surgical procedure for malignant nodules depended on the tumor stage and lymph node metastasis status. Ultrasonography could reveal nodule size, extraglandular invasion, and cervical lymph node metastasis. In our study, large nodules and those associated with obvious extraglandular invasion or lymphatic metastasis were treated using lobectomy or total thyroidectomy and central compartment or lateral neck dissection. Patients with invasion of the respiratory or digestive tract underwent surgery plus radioactive iodine treatment and radiotherapy. Patients with central lymph node metastasis were treated with total thyroidectomy and central compartment neck dissection. Prophylactic unilateral or bilateral central compartment neck dissection was performed for patients with advanced-stage papillary thyroid carcinoma (c3, T_4_ and cN1b). Patients with smaller lesions (T_1_, T_2_), non-invasive lesions, or cN_0_ papillary thyroid carcinoma, and most patients with follicular carcinoma underwent thyroidectomy with or without prophylactic central compartment dissection.

The accuracy of each classification in the diagnosis of benign and malignant nodules differed with nodule size. In our research, the thyroid nodules were divided into three groups based on diameter: ≤10 mm, >10 mm but ≤20 mm, and >20 mm. The diagnostic efficacy was higher for the larger nodules. For nodules measuring ≤10 mm, the EU-TIRADS had the highest SEN and NPV, while the ACR-TIRADS had the highest SPE, PPV, and AUC. In the other two groups, the Kwak-TIRADS had the highest SEN, NPV, and AUC, while the ACR-TIRADS had the highest SPE and PPV. All four risk-stratification systems performed well in the differential diagnosis of benign and malignant thyroid nodules.

Overall, the Kwak-TIRADS had the highest SEN, NPV, and ACC, while the ACR-TIRADS had the highest SPE and PPV. The Kwak-TIRADS significantly differed from the other three guidelines, but no significant differences were found among the other three guidelines. The Kwak-TIRADS may be the optimal classification system to differentiate between benign and malignant thyroid nodules.

There are several limitations to this study. First, selection bias was inevitable because of the retrospective study design and because patients were selected from the surgical department rather than the general population. Second, there may be inter-rater differences between the characteristics of thyroid nodules. The consistency between assessments performed by different physicians should be verified. Finally, the proportion of malignant nodules was high, and papillary thyroid carcinomas accounted for the majority of the malignant nodules, with few cases of other types of malignant nodules. Prospective studies with larger sample sizes may overcome this drawback.

In conclusion, all four risk-stratification systems provided effective stratification of malignancy risk for the diagnosis of thyroid nodules. The Kwak-TIRADS may be more suitable for the Chinese population, and is simple worthy of clinical application.

## Ethics Statement

This study was carried out in accordance with the recommendations of Declaration of Helsinki with written informed consent from all subjects. All subjects gave written informed consent in accordance with the Declaration of Helsinki. The protocol was approved by the Ethics committee of Gong Li Hospital.

## Author Contributions

YS and XF conceived and designed the experiments. JH, SW, MC, YW, and LG performed the experiments. YS and ML reviewed, analyzed, and classified the imaging data. XC provided basic information of all cases, and JD provided pathological results. YS wrote the paper.

### Conflict of Interest Statement

The authors declare that the research was conducted in the absence of any commercial or financial relationships that could be construed as a potential conflict of interest.

## References

[1] Gharib H Papini E Garber JR Duick DS Harrell RM Hegedus L. American association of clinical endocrinologists, American college of endocrinology, and associazione medici endocrinologi medical guidelines for clinical practice for the diagnosis and management of tyroid nodules-2016 update. Endocr Pract. (2016) 22:622–39. 10.4158/EP161208.GL 27167915

[2] Gharib H Papini E Paschke R Duick DS Valcavi R Hegedus L. American association of clinical endocrinologists, associazione medici endocrinologi, and European thyroid association medical guidelines for clinical practice for the diagnosis and management of thyroid nodules. Endocr Pract. (2010) 16(Suppl. 1):1–43. 10.4158/10024.GL 20497938

[3] Russ G Bonnema SJ Erdogan MF Durante C Ngu R Leenhardt L. European Thyroid association guidelines for ultrasound malignancy risk stratification of thyroid nodules in adults: the EU-TIRADS. Eur Thyroid J. (2017) 6:225–37. 10.1159/000478927 29167761 PMC5652895

[4] Singh Ospina N Brito JP Maraka S Espinosa De Ycaza AE Rodriguez-Gutierrez R Gionfriddo MR. Diagnostic accuracy of ultrasound-guided fine needle aspiration biopsy for thyroid malignancy: systematic review and meta-analysis. Endocrine. (2016) 53:651–61. 10.1007/s12020-016-0921-x 27071659

[5] Ross DS. Nonpalpable thyroid nodules–managing an epidemic. J Clin Endocrinol Metab. (2002) 87:1938–40. 10.1210/jc.87.5.1938 11994320

[6] Xing M Alzahrani AS Carson KA Viola D Elisei R Bendlova B. Association between BRAF V600E mutation and mortality in patients with papillary thyroid cancer. JAMA. (2013) 309:1493–501. 10.1001/jama.2013.3190 23571588 PMC3791140

[7] Zhang B Jiang YX Liu JB Yang M Dai Q Zhu QL. Utility of contrast-enhanced ultrasound for evaluation of thyroid nodules. Thyroid. (2010) 20:51–7. 10.1089/thy.2009.0045 20067379

[8] Xu JM Xu XH Xu HX Zhang YF Zhang J Guo LH. Conventional US, US elasticity imaging, and acoustic radiation force impulse imaging for prediction of malignancy in thyroid nodules. Radiology. (2014) 272:577–86. 10.1148/radiol.14132438 24689885

[9] Kwak JY Han KH Yoon JH Moon HJ Son EJ Park SH. Thyroid imaging reporting and data system for US features of nodules: a step in establishing better stratification of cancer risk. Radiology. (2011) 260:892–9. 10.1148/radiol.11110206 21771959

[10] Haugen BR Alexander EK Bible KC Doherty GM Mandel SJ Nikiforov YE. 2015 American thyroid association management guidelines for adult patients with thyroid nodules and differentiated thyroid cancer: the American thyroid association guidelines task force on thyroid nodules and differentiated thyroid cancer. Thyroid. (2016) 26:1–133. 10.1089/thy.2015.0020 26462967 PMC4739132

[11] Tessler FN Middleton WD Grant EG Hoang JK Berland LL Teefey SA. ACR thyroid imaging, reporting and data system (TI-RADS): white paper of the ACR TI-RADS committee. J Am Coll Radiol. (2017) 14:587–95. 10.1016/j.jacr.2017.01.046 28372962

[12] Wolinski K Szkudlarek M Szczepanek-Parulska E Ruchala M. Usefulness of different ultrasound features of malignancy in predicting the type of thyroid lesions: a meta-analysis of prospective studies. Pol Arch Med Wewn. (2014) 124:97–104. 10.20452/pamw.2132 24473342

[13] Cooper DS Doherty GM Haugen BR Kloos RT Lee SL Mandel SJ. Revised American Thyroid Association management guidelines for patients with thyroid nodules and differentiated thyroid cancer. Thyroid. (2009) 19:1167–214. 10.1089/thy.2009.0110 19860577

[14] Frates MC Benson CB Charboneau JW Cibas ES Clark OH Coleman BG. Management of thyroid nodules detected at US: society of radiologists in Ultrasound consensus conference statement. Radiology. (2005) 237:794–800. 10.1148/radiol.2373050220 16304103

[15] Horvath E Majlis S Rossi R Franco C Niedmann JP Castro A. An ultrasonogram reporting system for thyroid nodules stratifying cancer risk for clinical management. J Clin Endocrinol Metab. (2009) 94:1748–51. 10.1210/jc.2008-1724 19276237

[16] Park JY Lee HJ Jang HW Kim HK Yi JH Lee W. A proposal for a thyroid imaging reporting and data system for ultrasound features of thyroid carcinoma. Thyroid. (2009) 19:1257–64. 10.1089/thy.2008.0021 19754280

[17] Seo H Na DG Kim JH Kim KW Yoon JW. Ultrasound-based risk stratification for malignancy in thyroid nodules: a four-tier categorization system. Eur Radiol. (2015) 25:2153–62. 10.1007/s00330-015-3621-7 25680723

[18] Na DG Baek JH Sung JY Kim JH Kim JK Choi YJ. Thyroid imaging reporting and data system risk stratification of thyroid nodules: categorization based on solidity and echogenicity. Thyroid. (2016) 26:562–72. 10.1089/thy.2015.0460 26756476

[19] Russ G. Risk stratification of thyroid nodules on ultrasonography with the French TI-RADS: description and reflections. Ultrasonography. (2016) 35:25–38. 10.14366/usg.15027 26324117 PMC4701367

[20] Shin JH Baek JH Chung J Ha EJ Kim JH Lee YH. Ultrasonography diagnosis and imaging-based management of thyroid nodules: revised korean society of thyroid radiology consensus statement and recommendations. Korean J Radiol. (2016) 17:370–95. 10.3348/kjr.2016.17.3.370 27134526 PMC4842857

[21] Zayadeen AR Abu-Yousef M Berbaum K. JOURNAL CLUB: retrospective evaluation of ultrasound features of thyroid nodules to assess malignancy risk: a step toward TIRADS. AJR Am J Roentgenol. (2016) 207:460–9. 10.2214/AJR.15.15121 27352123

[22] Zhang J Xu H Zhang Y Xu J Liu C Guo L. Prospective validation of the thyroid imaging reporting and data system on thyroid nodules. Chin J Med Ultrasound. (2014) 167–71. 10.3877//cma.j.issn.1672-6448.2014.02.01526131184

[23] Grant EG Tessler FN Hoang JK Langer JE Beland MD Berland LL. Thyroid ultrasound reporting lexicon: white paper of the ACR thyroid imaging, reporting and data system (TIRADS) committee. J Am Coll Radiol. (2015) 12:1272–9. 10.1016/j.jacr.2015.07.011 26419308

