# Supplementary material for: Comparison of Different Risk-Stratification Systems for the Diagnosis of Benign and Malignant Thyroid Nodules
Source: Front Oncol. 2019 May 14;9:378. doi: 10.3389/fonc.2019.00378 (PMC6527759; doi:10.3389/fonc.2019.00378)
Supplement: Supplementary file 1 [file Table_1.DOCX]

**Supplementary Table S1.** Pairwise comparison of the four guidelines

| **Parameter** | **z statistic** | ***P* value** |
| --- | --- | --- |
| ACR vs. ATA | 1.694 | 0.0903 |
| ACR vs. EU | 1.170 | 0.2408 |
| ACR vs. Kwak | 3.405 | 0.0007 |
| ATA vs. EU | 0.952 | 0.3413 |
| ATA vs. Kwak | 5.748 | <0.001 |
| EU vs. Kwak | 5.485 | <0.001 |

ACR, 2017 American College of Radiology guidelines; EU, 2017 European Thyroid Association Guidelines; Kwak, 2011 TIRADS developed by Kwak *et al*.; ATA, 2015 American Thyroid Association Management Guidelines for Adult Patients with Thyroid Nodules and Differentiated Thyroid Cancer
